# Assessing a national policy on strengthening chronic care in primary care settings of a middle-income country using patients’ perspectives

**DOI:** 10.1186/s12913-021-06220-x

**Published:** 2021-03-12

**Authors:** Wichai Aekplakorn, Paibul Suriyawongpaisal, Samrit Srithamrongsawadi, Phanuwich Kaewkamjonchai

**Affiliations:** Department of Community Medicine, Faculty of Medicine, Ramathibodi Hospital, Mahidol University, Ratchathewi, Bangkok, 10400 Thailand

**Keywords:** Primary care, PACIC, Chronic care, NCD, Policy, A middle-income country, Patients’ perspective

## Abstract

**Background:**

To improve care for patients with chronic diseases, a recent policy initiative in Thailand focused on strengthening primary care based on the concept of Chronic Care Model (CCM). This study aimed to assess the perception of patients about the health care services after the implementation.

**Methods:**

We conducted a cross-sectional survey of 4071 patients with hypertension and/or diabetes registered with 27 primary care units and 11 hospital non-communicable diseases (NCDs) clinics in 11 provinces.

The patients were interviewed using a validated questionnaire of the Patient Assessment of Chronic Illness Care. Upgraded primary care units (PCUs) were ordinary PCUs with the multi-professional team including a physician. Trained upgraded PCUs were upgraded PCUs with the training input. Structural equation modeling was used to create subscale scores for CCM and 5 A model characteristics. Mixed effect logistic models were employed to examine the association of subscales (high vs low score) of patient perception of the care quality with type of PCUs.

**Results:**

Compared to hospital NCD clinics, ordinary PCUs were the best in the odds of receiving high score for every CCM subscale (ORs: 1.46–1.85; *p* < 0.05), whereas the trained upgraded PCUs were better in terms of follow-up (ORs:1.37; *p* < 0.05), and the upgraded PCU did not differ in all domains. According to the 5 A model subscales, patient assessment also revealed better performance of ordinary PCUs in all domains compared to hospital NCD clinics whereas upgraded PCUs and trained upgraded PCUs did so in some domains. Seeing the same doctor on repeated visits (ORs: 1.82–2.17; *p* < 0.05) or having phone contacts with the providers (ORs:1.53–1.99; *p* < 0.05) were found beneficial using CCM subscales and the 5A model subscales. However, patient assessment by both subscales did not demonstrate a statistically significant association across health insurance status.

**Conclusions:**

The policy implementation might not satisfy the patients’ perception on quality of chronic care according to the CCM and the 5A model subscale. However, the arrangement of chronic care with patients seeing the same doctors or patients having telephone contact with healthcare providers may satisfy the patients’ perceived needs.

**Supplementary Information:**

The online version contains supplementary material available at 10.1186/s12913-021-06220-x.

## Background

Low- and middle-income countries (LMICs) do not only face a disproportionately heavy burden of chronic non-communicable diseases (NCD), but also have difficulties in scaling-up service delivery models such as the Chronic Care Model (CCM), a well-structured approach to caring for patients with chronic diseases, which has proven effective in high-income countries [1, 2]. So far, empirical data on the application of CCM or other strategies to address healthcare needs of patients with NCD in primary care settings of LMICs has primarily been confined to pilot scale or individual studies [3, 4].

CCM is a well-accepted approach to improve the quality of care of chronic diseases. It comprises six domains: community, health system, self-management support, delivery system design, decision support and clinical information system [5]. The challenges of chronic care include a health system that incorporates adequate service components to provide quality of care such as case detection, identification of risk groups and patients, treatment and long-term follow-up, promotion of treatment adherence, and life-style modification [2].

To meet the need for better chronic care, studies have shown that financial and infrastructural resources alone are insufficient [2]. LMIC health systems also require human and institutional capacity strengthening to improve the effectiveness, quality, distribution, and continuity of care through smart designs and use of technology [6, 7]. In low- and middle-income country settings, adapting disease guidelines requires non-physician clinicians to deliver care and to ensure effective implementation of standardised protocols for diagnosis, treatment, and monitoring [7, 8].

Despite the presence of universal healthcare coverage (UHC) in Thailand over the past two decades, provision of chronic care for patients with hypertension and/or diabetes in primary care settings has faced a challenge of shortage of health care workforce especially nurses [9]. Furthermore, the policymakers have been increasingly aware of the importance of training needs for quality improvement of chronic care [10]. To address the limitations, the Ministry of Public Health has taken a policy initiative to strengthen the chronic care for patients with diabetes or hypertension with a two-pronged strategy: a) allocation of a family physician and multidisciplinary health professional as a team to primary care settings at subdistrict with a well-defined population of approximately 10,000; and b) training of the team to deliver healthcare based on the concept of CCM. Recently, a systematic review has confirmed that Self-management Support is the most frequent CCM intervention which is associated with statistically significant improvements, predominately for diabetes and hypertension [11]. To address the knowledge gap of up scaling the chronic care, assessments of patients’ perspective is a critical input requirement. In the present study we aimed to assess the perception of patients about their health care services after the policy implementation using the validated questionnaire of the Patient Assessment of Chronic Illness Care (PACIC+) [12].

## Methods

### Settings

Our study was undertaken in the context of public healthcare systems under supervision of the MOPH and local authorities which cover around 80% of the whole population. In light of the increasing awareness of the complexity of implementation research, the present study adopted the multilayered approach ranging from individual or family to local or district level, to take account of the contextual influence on policy implementation. At healthcare setting layer, we focused on primary care settings comprising NCD clinics in referral (tertiary to secondary care) hospitals, and primary care units (PCU) at subdistrict level of both municipality and non-municipality. Together, these healthcare facilities were considered healthcare organisation layer nested within geographical layer of local administrative structure.

In each district, NCD clinics and PCUs form a referral network of healthcare facilities providing outpatient care for patients with chronic or acute conditions (only applied to PCUs). In general, each NCD clinic is staffed with a doctor, 3–6 nurses, and 1–4 public health workers full-time. Part-time staff of each NCD clinic varies considerably across districts in types and number. Each NCD clinic might be staffed with part-time staff as follows: 0–4 dentists or dental hygienists, 0–2 pharmacists, 0–1 physiotherapist, and 0–1 dietitian. Similarly, each PCU might be staffed with 0–1 doctor, 1–2 nurses, and 1–6 public health workers full-time. Part-time staff of each PCU also vary considerably across subdistricts in types and number. At individual level, personal characteristics such as demographic profiles, health insurance status/scheme, and types of chronic conditions, were considered to reflect their influence on the achievement of the policy interventions, based on the principle of integrated and people-centered health services [13].

### Policy interventions

To improve care for patients with chronic diseases, a recent policy initiative in Thailand has focused on strengthening primary care with a two-pronged strategy. Since 2016, the first strategy of allocation of family physicians and teams has been applied to an accumulated number of 1137 primary care units (PCU) or 11.6% of the total of 9777 in 74 provinces [http://pcc.moph.go.th/pcc/dashboard/?p=teamCount_rpt]. A physician trained in family medicine and new medical equipment (such as ultrasonography, ECG monitor) were distributed to each selected PCU dubbed “upgraded PCU”. The physician was assigned to provide full-time clinical services for 3–5 days a week to the upgraded PCU in addition to outpatient care services in the referral hospital including NCD clinics. Due to a time lag in implementing the training, a number of upgraded PCUs had not received the training at the time of our study (started 2 months after the first batch (*N* = 13) of trained upgraded PCUs). In contrast, patients seeking care at ordinary PCUs have only 1 day per week to receive care from the team and a physician with or without training in family medicine. Ordinary PCUs did not receive any additional resources since they were expected to refer patients with repeatedly uncontrolled hypertension or diabetes to upgraded PCUs or hospital NCD clinics.

In July 2019, the MOPH implemented the second strategy by providing training program to 13 of 21 upgraded PCUs in 11 provinces. These provinces comprised 21 clusters of PCUs, each with 1 upgraded PCU as a node of the cluster, and another 0–1 ordinary PCUs. The total number of ordinary PCUs in the implemented district of these provinces was 8. From each of the 13 upgraded PCUs, the head and 2–3 clinicians attended two consecutive training workshops (1 and a half days each). The first one started with a didactic lecture addressing the concepts of the strategy and tools for translating the concepts into practices i.e., system thinking and design thinking [14, 15]. Two small group sessions followed the lecture, to discuss experiences and ideas related to the translation of the knowledge tailored to specific settings. Reading materials focusing on WHO’s Integrated People-centered Health Service (IPCHS) [13] and CCM [1] were shared with the participants. The second workshop followed 1 month after the first to explore the feasibility and barriers of implementing the strategy making use of the participant experiences. The participants were expected to transfer the knowledge and skills to the rest of the team members in each upgraded PCU. To ensure fidelity of the implementation theory, follow up support and encouragement throughout the study period were carried out by two implementation support practitioners. They paid a visit to each team of participants which aimed at activating implementation-relevant knowledge, skills, and attitudes, and to operationalise and apply these in the context of those participants. In doing so, they aimed to trigger both relational and behavioral outcomes. For instance, the application of the concept of risk stratification of the patients was encouraged, in order to customise clinical transactions according to the needs of specific patients, instead of treating all patients similarly which usually results in superficial provider-patient dialogue, and refilling medications over a period of just 3–5 min for each patient. Nevertheless, there was no systematic check of the fidelity.

### Rationale of study design

In real world practices, evidence-based interventions (EBIs) are implemented in complex, multi-faceted and dynamic environments, which arguably means that the same intervention would rarely work in the same way in different contexts [16]. A three-dimensional framework has emphasised the relationship between: (a) the type of the evidence being used, (b) the ability of the context to cope with change and (c) the facilitation needed for a successful change process. Therefore, while the tools and strategies used to implement an intervention are important, the context of implementation equally matters. Based on this rationale, we consulted stakeholders (policymakers, a major healthcare payer, healthcare providers and research funder) to explore their concerns and insights related to the policy implementation and assessment. Given the timing of the assessment 2 months after the implementation), most stakeholders put major emphasis on feasibility of the implementation and hence preferred demonstration of best-case scenario as a representative picture. As a result, we ended up with the study design and sampling as follows. With this approach we were aware of potential bias by the choice of samples that were more likely to produce sizeable outcomes. Nonetheless, if the opposite evidence were found, it could support the argument of policy implementation failure given the lack of fidelity check as mentioned.

The study design and sampling served assessment needs in terms of contextual assessment and assessment of the policy interventions. The outcome of interest in our study was patients’ assessment of the quality of care, which is a key component of overall quality of care, according to the WHO European Framework for action on integrated health services delivery [17]. Our outcome of choice was considered appropriate for the timing of assessment which started just 2 months after the training, to enable the team of clinicians of each selected upgraded PCU to organise patient-centered care for diabetes or hypertension. In fact, there is another tool for assessing chronic care using provider perspective (Assessment of Chronic Illness Care), but it has been proved inappropriate for widespread use due to over-reporting problem [18]. We expected the findings at the initial stage of the policy implementation would be a guiding light on whether the policy was heading in the right direction rather than assessing its effectiveness.

### Patient survey

#### Population and samples

**Fig. 1:**
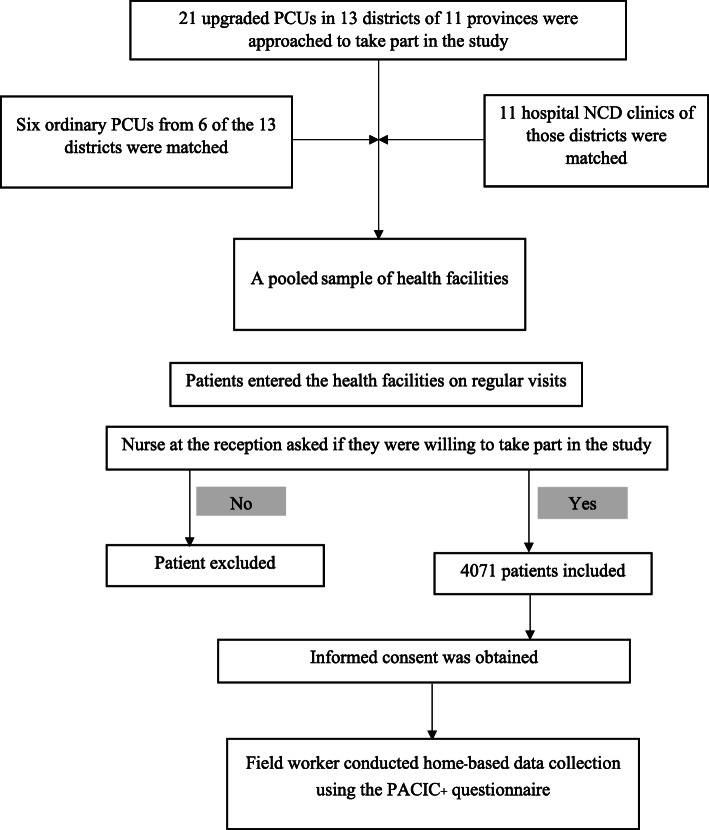
Flow of activities on sample selection and data collection

To facilitate assessment of the training effects, trained upgraded PCUs were matched to other health facilities without training in the same district: hospital NCD clinic, untrained upgraded PCUs and ordinary PCUs. The number of PCUs and hospital NCD clinics in the same districts which agreed to participate included: 21 upgraded PCUs (13 trained upgraded PCUs, 8 untrained upgraded PCUs), 6 ordinary PCUs and 11 hospital NCD clinics (Fig. 1). The hospital NCD clinics were included since they were supposed to care for the most complicated patients (single end-organ involvement) referred from PCUs. Upgraded PCUs were supposed to care for less complicated patients (repeated poor control of BP or FBS/HbA1c) regardless of training exposure. Ordinary PCUs were assigned to take care of patients in well-controlled status without complications. All patients with diabetes or hypertension who made regular visits to these participating health facilities were asked to participate in the study.

In total, 4071 patients gave informed consent and were interviewed at home using the PACIC+ questionnaire by trained field workers during September 2019.

### Data collection

The Chronic Care Model (CCM) identifies six domains that are essential to provide good quality of care for chronic illnesses: the community, the health system, self-management support, delivery system design, decision support and clinical information systems. High quality care models e.g., patient centered medical home and chronic care model had been proven effective in improving diabetes care or care for patients with multiple chronic conditions. In a similar vein, 5A model has achieved widespread acceptance and reflects the core elements of patient-centered care in chronic diseases. The “5A” model represents an evidence-based approach to induce a behavioral change. The key elements are: assessment of present behaviour (Assess), patient counselling (Advise), collaborative agreement with the patient about realistic goals (Agree), assisting the patient during her/his lifestyle changes (Assist) and frequent follow-ups (Arrange). Another CCM evaluation tool is the Patient Assessed Chronic Illness Care (PACIC). The PACIC is a 20-item survey which measures the patient’s perceived quality of care retrospectively for 6 months. The PACIC+ additionally addresses the evidence-based 5A model for behavioral changes and was developed in order to fill the same gap for the 5A model that existed for the CCM. The 20 items from the PACIC are complemented by another 6 items in order to improve content validity and to enable the assessment of factors related to the 5A model, which is a counseling model for behavioral changes.

In our study, the PACIC+ questionnaire was adopted from the Thai version of the Patient Assessment of Chronic Illness Care (PACIC) validated in outpatient clinic of a university hospital in Thailand with high reliability (Cronbach’s alpha per subscale varied from 0.58 to 0.81 and that of the summary scores were 0.89 and 0.91) [12]. The PACIC+ contains 26 items. Twenty items are from the original PACIC, which measure different parts of the CCM, and an additional 6 items assess the 5A Model. Each item asks the patients to evaluate the care they have received in the past 6 months on a 5-point scale: 1 (Almost never), 2 (Usually not), 3 (Sometimes), 4 (Mostly) and 5 (Almost always). It takes approximately 5–10 min to complete. The items of the PACIC+ are grouped into CCM subscales and 5A Model subscales. The CCM subscales constitute: Patient Activation (items 1–3); Delivery System (items 4–6); Goal Setting (items 7–11); Problem solving (items 12–15); and Follow-up (items 16–20). The 5A Model consists: Assess (items 1,11, 15, 20, 21); Advise (items 4, 6, 9, 19, 24); Agree (items 2, 3, 7, 8, 25); Assist (items 10, 12, 13, 14, 26); and Arrange (items 16, 17, 18, 22, 23).

In our study, there were 3 sections in the questionnaire: 1) personal information of the respondents such as demographic profiles, health insurance status; 2) perceptions of their interactions with providers, and 3) PACIC+ items. A supplementary file provided details of the 3 sections [see Additional file 1]. In brief, the personal information consisted of demographic profile, type of chronic conditions, duration of the conditions and health insurance status. Section 2 explored perception about: channel of contact with providers, and receiving care from the same provider on repeated visits.

### Statistical analysis

We constructed 2 sets of latent variables as subscales from the PACIC+ items to reflect the components of the chronic care model and 5 A model. The subscales for components of the chronic care model (CCM) included patient activation, delivery system, goal setting, problem solving and follow-up and for the 5 A model components included assess, advise, agree, assist, and arrange and those were created using structural equation model [19, 20]. Confirmatory factor analysis was performed using structural equation modeling to evaluate the fitness of the data to the PACIC+ scale structure. The extent to which the items loaded on to the hypothesised variables and the correlation [see Table A1, A2 in Additional file 2] were examined.

For CCM subscales, almost all the factors have factor loadings of 0.60 or greater, and only 4 items had standardised factor loadings less than 0.6. The goodness of fit for the overall model was moderate, and the value of RMSEA and CFI were 0.092 and 0.863, respectively. For 5A the overall goodness of fit was slightly lower than that of CCM. Percentage of categorical variables and mean (standard deviation) by type of primary care setting were calculated and tested for statistical significance with chi-square test and t-test respectively. For comparison of subscale scores across types of primary setting, median and interquartile range were calculated and compared with Kruskal Wallis test. Each subscale representing a composite variable for each set of PCIC+ indicators was used in the association analysis as they accounted for the variance and covariance of the variables in the respective set.

To examine the individual factors and types of primary care setting that were associated with patient perception measures, PACIC, each subscale was categorised into binary variables cut at percentile 75th (0 = low score, and 1 = high score) and treated as the outcome variable. Chi-square’s test was performed to explore the association between each independent variable and each outcome and variables that provided *p*-value of less than 0.10 were included in the multivariate regression analysis. The multilevel regression analysis was considered, as the first level was individual, and the second level was the primary care cluster. Mixed effects logistic regression model was used to examine the association between each subscale and the explanatory variables with a random intercept for “primary care unit (PCU)” level to take into account the correlation among patients in the same PCU. Independent variables that were in the multivariable regression analysis included individual level: sex, age, education (primary, secondary and bachelor), chronic diseases (DM, HT, and both), duration of the chronic disease condition, knew about family doctor (yes/no) or health care provider’s name (yes/no), type of contact channel (no, mobile phone, Line application, and others), seeing same doctor on repeated visits, (yes/no) and health insurance status/scheme (universal health, social security, civil servant, and others) and type of primary care setting (trained PCU, upgraded PCU, ordinary PCU, and NCD clinic in hospital). Odds ratio and 95% CI were calculated and reported. Stata version 16 (StataCorp. 2019. College Station, TX) was used for the statistical analysis.

## Results

### Baseline characteristics of respondents

Most of the 4071 respondents were: female (73%) aged 59 ± 21.25 on average with primary level of education (81%). Patients in untrained upgrade were significantly younger than other settings. Almost half (49%) of them were hypertensive and 38% had both hypertension and diabetes with average duration of 7–10 years, and patients with diabetes and hypertension were highest in hospital clinics, (Table 1). The majority of them reported a well-controlled status (66%). Eighty two percent were covered with universal coverage scheme, the biggest public health insurance with the largest population coverage. All the subscale scores for components of CCM and 5A for the trained upgraded ordinary PCUs were highest, followed by the trained upgraded PCUs scores obtained in the other 3 types of healthcare facilities (Table 1).

**Table 1:** Health facility types and individual patients’ characteristics (*N* = 4071)

	N	Hospital NCD clinic ***n*** = 1160	Trained upgraded PCU ***n*** = 1822	Upgraded PCU ***n*** = 757	Ordinary PCU ***n*** = 332	***p***-value
Female	2980	74.66	73.48	72.2	69.49	0.26
Age, mean (SD)	4071	58.73 (22.21)	60.76 (18.11)	53.17 (25.63)	59.5 (20.58)	< 0.001
**Educational attainment**						
< =Primary	331	79.31	80.01	85.83	89.76	< 0.001
Secondary	633	17.4	17.06	12.58	8.43	
Bachelor	109	3.29	2.93	1.59	1.81	
**Chronic diseases**						
Diabetes	553	16.47	13.01	12.15	9.94	< 0.001
Hypertension	1988	35.00	52.94	57.20	55.72	
Diabetes and hypertension	1529	48.53	34.05	30.65	34.34	
Duration of disease, mean (SD)	4071	9.39 (7.51)	8.34 (7.07)	7.39 (6.87)	7.08 (6.42)	0.002
Knew about family doctor name	2306	64.51	50.58	59.18	57.23	< 0.001
Knew provider name	2418	49.87	69.48	50.53	57.83	< 0.001
**Communication Channel**						
No	2952	73.85	67.00	77.75	89.09	< 0.001
Phone	849	19.65	25.74	16.16	9.70	
Line	47	1.13	1.21	1.19	0.91	
Others	210	5.37	6.05	4.90	0.30	
Same doctor	2699	49.40	71.06	72.26	85.84	< 0.001
**Health Insurance status**						
Universal Coverage Scheme; UC	3353	74.91	82.55	89.83	90.36	< 0.001
Social security scheme; SSS	174	7.16	2.58	3.83	4.52	
Civil Servant Medical Benefit Scheme; CSMBS	379	14.48	9.06	5.02	2.41	
Others	165	3.45	5.82	1.32	2.71	
**CCM score, Median (IQR)**						
Patient activation	4071	−0.04 (1.62)	0.14 (1.13)	− 0.004 (1.49)	0.21 (1.40)	< 0.001
Delivery	4071	0.01 (1.44)	0.16 (0.89)	−0.05 (1.28)	0.23 (1.18)	
Goal setting	4071	−0.03 (1.46)	0.16 (0.99)	−0.04 (1.46)	0.22 (1.28)	
Problem solving	4071	0.01 (1.25)	0.15 (0.83)	0.001 (1.10)	0.20 (0.97)	
Follow-up	4071	−0.08 (1.38)	0.05 (1.40)	−0.11 (1.60)	0.03 (1.57)	
**5A score, median (IQR)**						
Assess	4071	−0.08 (1.38)	0.12 (1.08)	−0.08 (1.43)	0.14 (1.11)	
Advise	4071	−0.03 (1.40)	0.14 (0.93)	−0.07 (1.38)	0.23 (1.13)	
Agree	4071	−0.02 (1.35)	0.16 (0.95)	−0.04 (1.33)	0.20 (1.13)	
Assist	4071	−0.04 (1.58)	0.15 (1.07)	−0.05 (1.62)	0.20 (1.28)	
Arrange	4071	−0.08 (1.36)	0.14 (1.00)	−0.03 (1.38)	0.20 (1.02)	

### Relationships between the policy interventions, context, and outcome

Using mixed effect modeling adjusted for age, sex, education, and duration of the chronic conditions; we found association between CCM subscales and individual patient characteristics or health facility type (healthcare setting) as follows.

Across the 5 subscales of CCM, ORs for patients attending ordinary PCU (OPCU) responded with high scores which were 2–4 times higher compared to those for patients attending hospital NCD clinics (Table 2). This was also the case for patients exposed to different service delivery components regardless of healthcare settings: seeing the same doctor on repeated visits (ORs: 1.82–2.17) or having phone contacts of the providers (ORs: 1.53–1.99). Similarly, irrespective of healthcare settings, patients with hypertension were less likely to do so as compared to those with diabetes for 2 subscales: goal setting (OR 0.84) or follow-up (OR 0.71). Patients with both diabetes and hypertension were more likely than those with diabetes to make such a report of problem solving/contextual counseling (OR 1.24). In contrast, there was no statistically significant association between health insurance status and patients’ reports of any subscale.

**Table 2:** Association between predictors and CCM subscales according to mixed effect modeling adjusted for age, sex, education, and duration of chronic conditions

	Patient activation		Delivery		Goal setting		Problem solving		Follow-up	
	Odds Ratio	95% Conf.	Odds Ratio	95% Conf.	Odds Ratio	95% Conf.	Odds Ratio	95% Conf.	Odds Ratio	95% Conf.
**PCU type**										
Hospital clinic	1	1	1		1		1		1	
Trained upgraded PCU	1.09	(0.90, 1.32)	1.17	(0.97, 1.40)	1.18	(0.98, 1.42)	1.06	(0.88, 1.27)	1.37*	(1.14, 1.66)
Upgraded PCU	1.29	(1.00, 1.67)	1.18	(0.92, 1.52)	1.22	(0.94, 1.57)	1.16	(0.90, 1.48)	1.05	(0.81, 1.36)
Ordinary	1.71*	(1.25, 2.32)	1.60*	(1.18, 2.17)	1.85*	(1.36, 2.52)	1.63*	(1.20, 2.20)	1.45*	(1.06, 1.98)
**Chronic conditions**										
DM	1	1	1		1		1		1	
HT	0.85	(0.68, 1.05)	0.94	(0.76, 1.16)	0.84	(0.68, 1.04)	0.97	(0.78, 1.19)	0.71*	(0.57, 0.88)
DM + HT	1.23	(0.99, 1.53)	1.23	(0.99, 1.53)	1.18	(0.94, 1.47)	1.24	(1.00, 1.54)	1.09	(0.87, 1.36)
**Communication channels**										
No	1.00									
Phone	1.99*	1.66, 2.38)	1.86*	(1.56, 2.22)	1.95*	(1.63, 2.34)	1.86*	(1.56, 2.21)	1.53*	(1.28, 1.83)
Line	1.10	(0.59, 2.08)	1.32	(0.71, 2.45)	1.46	(0.78, 2.73)	1.19	(0.64, 2.21)	1.56	(0.84, 2.91)
Others	0.77	(0.56, 1.05)	0.78	(0.57, 1.07)	0.68*	(0.49, 0.94)	0.86	(0.63, 1.18)	0.48*	(0.34, 0.67)
**Seeing same doctors**										
No	1	1	1		1		1		1	
Yes	1.96*	(1.68, 2.30)	1.85*	(1.58, 2.16)	1.98*	(1.69, 2.33)	1.82*	(1.56, 2.12)	2.17*	(1.85, 2.54)
**Health insurance status**										
UC	1.00									
SSS	1.14	(0.81, 1.61)	1.18	(0.84, 1.66)	1.03	(0.73, 1.47)	1.22	(0.87, 1.71)	0.94	(0.65, 1.36)
CSMBS	0.94	(0.72, 1.23)	1.01	(0.78, 1.31)	0.97	(0.74, 1.27)	0.90	(0.70, 1.17)	0.94	(0.72, 1.36)
Others	1.06	(0.74, 1.52)	1.13	(0.79, 1.60)	1.12	(0.78, 1.60)	1.08	(0.76, 1.53)	1.03	(0.71, 1.48)

All models were adjusted for age, sex, education, duration of illnesses; * *p* < 0.05; *UC* Universal Coverage Scheme, *SSS* Social Security Scheme, *CSMBS* Civil Servant Medical Benefit Scheme

Considering care for behavioral change, the analysis revealed association between the 5A model subscales and individual patient characteristics or healthcare setting as follows. Across the 5A subscales, ORs for patients attending ordinary PCU responded with high scores which were 1.40–2.00 times higher compared to those for patients attending hospital NCD clinics (Table 3). Irrespective of healthcare settings, patients who met the same doctor on repeated visits reported a higher OR than those who did the opposite (OR: almost 2). Patients having mobile phone contacts of the providers also reported a higher OR than those who did not (ORs: 1.79–2.14).

**Table 3:** Association between predictors and the 5 A model subscales according to mixed effect modeling adjusted for age, sex, education, and duration of chronic conditions

	Assess		Advice		Agree		Assist		Arrange	
	Odds Ratio	95% Conf.	Odds Ratio	95% Conf.	Odds Ratio	95% Conf.	Odds Ratio	95% Conf.	Odds Ratio	95% Conf.
**PCU type**										
Hospital clinic	1	1	1		1		1		1	
Trained upgraded PCU	1.17	(0.97, 1.42)	1.12	(0.93, 1.35)	1.26	(1.05, 1.52)	1.33*	(1.10, 1.60)	1.35*	(1.12, 1.63)
Upgraded PCU	1.04	(0.81, 1.35)	1.21	(0.94, 1.56)	1.35*	(1.05, 1.74)	1.46*	(1.13, 1.88)	1.33*	(1.03, 1.73)
Ordinary	1.48*	(1.09, 2.02)	1.84*	(1.36, 2.51)	1.86*	(1.37, 2.52)	1.82*	(1.34, 2.48)	2.10*	(1.54, 2.86)
**Chronic conditions**										
DM	1	1	1		1		1		1	
HT	0.87	(0.70, 1.08)	0.85	(0.68, 1.05)	0.92	(0.74, 1.13)	0.82	(0.66, 1.01)	0.86	(0.70, 1.07)
DM + HT	1.20	(0.96, 1.49)	1.18	(0.95, 1.47)	1.26*	(1.01, 1.56)	1.14	(0.92, 1.42)	1.18	(0.95, 1.47)
**Channel of communications**										
No	1	1	1		1		1		1	
Phone	2.11*	(1.76, 2.53)	1.97*	(1.65, 2.36)	1.79*	(1.50, 2.14)	2.00*	(1.67, 2.40)	2.14*	(1.78, 2.56)
LINE application	1.33	(0.71, 2.47)	1.43	(0.77, 2.66)	1.45	(0.78, 2.69)	1.44	(0.77, 2.67)	1.48	(0.80, 2.76)
Others	0.65	(0.47, 0.90)	0.74	(0.54, 1.02)	0.70*	(0.51, 0.96)	0.73	(0.53, 1.01)	0.69*	(0.50, 0.95)
**Seeing the same doctor**										
No	1	1	1		1		1		1	
Yes	2.07*	(1.76, 2.42)	1.96*	(1.67, 2.29)	1.87*	(1.60, 2.18)	1.98*	(1.68, 2.32)	1.91*	(1.63, 2.24)
**Health insurance status**										
UC	1	1	1		1		1		1	
SSS	0.96	(0.67, 1.37)	1.18	(0.84, 1.67)	1.23	(0.87, 1.74)	1.06	(0.74, 1.50)	0.97	(0.68, 1.39)
CSMBS	1.02	(0.78, 1.32)	0.97	(0.75, 1.26)	1.12	(0.87, 1.46)	1.05	(0.80, 1.36)	1.02	(0.78, 1.33)
Others	0.85	(0.59, 1.22)	0.96	(0.67, 1.38)	1.03	(0.72, 1.47)	1.04	(0.72, 1.49)	0.92	(0.64, 1.32)

All models were adjusted for age, sex, education, duration of illnesses; * *p* < 0.05; *UC* Universal Coverage Scheme, *SSS* Social Security Scheme, *CSMBS* Civil Servant Medical Benefit Scheme

In the opposite way, patients with other contact channels were less likely to do so (ORs: 0.65–0.74) regardless of healthcare settings. There was no statistically significant association between health insurance status and patients’ reports of any 5A subscale.

## Discussion

According to patients’ perspective, the present study found that the patients’ perspective on quality of care at ordinary PCUs was superior to that at other healthcare facilities regardless for all the components of CCM and 5A model. In the following we explored plausible explanations for this finding.

### The training intervention and allocation of family physicians

In contrast to other studies in developed country settings, our findings did not support the training benefits of primary care providers on caring for patients with the chronic conditions using CCM. Thom et al., using randomised controlled-trials; RCT, demonstrated improved PACIC scores at 12 months from baseline (mean score: 3.82 vs 3.13; *p* < .001) and a significant difference in total PACIC score against usual care among low-income patients with poorly controlled diabetes, hypertension or dyslipidemia receiving care from trained health coaches in a US healthcare setting [21]. Their training was more intensive in terms of duration (40 h over 6 weeks) with specificity of clinical skills such as active listening and nonjudgmental communication; as well as helping with self-management skills as compared to that of our study context. Likewise, an upgraded study of clinical trial involving 57 patients with uncontrolled diabetes in a primary care setting in Brazil, showed outcome improvement over 6 months including PACIC score (33 to 68, *p* < 0.001) after repeated motivational interviews (MI) delivered by trained community health agents (CHA) on top of usual care [22]. In comparison to the training in our study, a distinctive feature of the training in this Brazilian study was ongoing assessment and feedback on MI skills of the CHA using a standardised tool (a fidelity checklist adapted from the “1-Pass Coding System for Motivational Interviewing”). The lack of fidelity check and feedback in our study settings might contribute to inconsistency of the implementation among trained upgraded PCUs which hence diluted the magnitude of associations.

Looking at the allocation of family physicians as an integral part of the policy intervention, those ORs discussed above also indicated insufficient evidence to support the expected effect of this policy component upon the quality of care.

Patients seeking care at ordinary PCUs have only 1 day per week to receive care from the team and a physician with or without training in family medicine. Ordinary PCUs did not receive any additional resources since they were expected to refer patients with repeatedly uncontrolled hypertension or diabetes to upgraded PCUs or hospital NCD clinics.

### The ability of the context to cope with change

In real world practices, evidence-based interventions (EBIs) are implemented in complex, multi-faceted and dynamic environments, which arguably means that the same intervention would rarely work in the same way in different contexts [16]. In our study context and literature, we considered three contextual factors with potential to influence the effect of the policy interventions as follows.

### Workforce-workload imbalance

Apart from the apparent limitations in the training approach identified by our study, the imbalance of health workforce against workload could be a major barrier to scaling-up quality improvement of chronic care [2, 23]. Using a multi-professional projection approach for Thailand, Pagaiya N et al. highlighted a severe shortage of nurses in year of 2026 whereas the supply of doctors, pharmacists, and physiotherapists is likely to be in surplus [9]. In primary care settings, the study identified the proportion of workload as 100% for nurses and 20% for doctors or other healthcare professionals. Hence, the shortage of nurses (not addressed by the policy) might explain the difficulty to improve quality of care for chronic diseases especially for those upgraded PCUs with higher number of registered population (10,000 per PCU) than that of ordinary PCUs (< 10,000 per PCU). This notion is supported by bigger ORs for the PACIC scores or 5A-model scores reported by the patients seeking care at ordinary PCUs than those at other facilities (Tables 2 and 3). The patients in the ordinary PCUs were more likely to spend less time than for those in the upgraded PCUs and hospital clinics due to fewer number of patients and less complicated condition. This might have great impact on their perception towards the care which they received.

In effect, no statistically significant association (*p* > 0.05) was found in a linear regression analysis of proportion of well-controlled DM or HT against ratio of nurse to population using clinical datasets of all health facilities in the main report of our study [24]. Similar findings were found in case of doctor-to-population ratio vs proportion of well-controlled DM or HT. These findings indicated the allocated number of family physicians (1 for every 10,000 population) could not match the workload and has not addressed the more obvious deficit of nursing stafs in primary care settings. In this respect, our study provided an early signal to policymakers regarding the need to consider focusing on allocating more nurses to primary care settings taking into account their workload which does not only correlate to the number of patients, but also to type of patient case-mix. The findings on smaller ORs, indicating lower quality of care, for upgraded PCUs versus those of ordinary PCUs attest a need to take account of the increasing demand from complicated patients referred from ordinary PCUs to upgraded PCUs and hospital NCD clinics.

### Mobile communication tools…a system enabler

Self-management support in chronic care could be enhanced by mobile communication tools such as telephone or online applications as indicated by accumulated evidence from randomised controlled-trials or systematic reviews [25, 26]. Our study provided evidence indicating the benefit of mobile telephone to support self-management of chronic care on a large scale (Tables 2 and 3). Compared to patients without any mobile contact channels, those with mobile phone contacts were more likely to give high to highest PACIC scores or 5A model scores, ORs ranging from 1.8–2.1, *p* < 0.05. In developed countries reported figures of citizens lacking basic digital skills in terms of digital literacy were around 40% in Europe and the U.S. [27] Based on our findings, it is implicated that mobile phone should be the first choice for the application of mobile information and communications technology; with ICT to support self-management in chronic care.

### The effects of patient–provider relationships and UHC

Better patient–provider relationships improve health and quality of care. The patient–provider relationship improves patients’ confidence in self-management of chronic conditions [28]. In our study context, we paid attention to the arrangement for patients to see the same doctors on repeated visits. Seeing the same provider helps patients develop a better relationship and make clinical decisions in a way that they prefer [29, 30].

With ORs close to 2 for the patients seeing the same doctors on repeated visits as compared to those without (*p* < 0.05) based on PACIC scores or 5A model scores, our study supported the importance of patient–provider relationships viewed from patients’ perspective.

Finally, with regard to equitable access to quality chronic care, our study findings of no association between health insurance status and the scores on PACIC or 5A model scale (ORs: 0.70–1.93; *p* > 0.05) render support to the effect of universal healthcare coverage in filling the inequity gaps. Nonetheless, concern about inadequacy in the power of tests could not be excluded.

### Implications

In accordance with the patients’ perspective, policymakers might find the training approach insufficient for strengthening chronic care at primary care setting due to the overburdened service load. The failure might also indicate a need to incorporate fidelity check into any training program dealing with chronic care aimed at addressing the complex healthcare needs. In addition, PACIC+ might be useful to assess and monitor the progress in nationwide implementation of the chronic care development in primary care settings.

### Limitations and strengths

With female patients overrepresented in our study, the results could hardly be applied to male patients. Causal inference is problematic given the cross-sectional design of our study. We did not account for conventional patient outcomes such as blood pressure, HbA1c, and adherence to medications. Despite the limitations, the sampling design involving primary care facilities in vast areas and the large number of respondents enabled us to assess the possibility of scaling-up policy interventions on quality improvement of chronic care using the validated standardised tools (the PACIC+) in a middle-income country with UHC. Given the paucity of evidence like this in a middle-income country setting, our study has made an important contribution to fill the knowledge gaps in scaling-up evidence-based approaches to strengthen the chronic care model in primary care settings of middle-income countries with UHC.

## Conclusions

Under the context of primary care in which shortage of nurses, a key clinical personnel, still exist; the allocation of family physicians in PCUs with or without adequate training of the primary care team may not satisfy the patients’ perception on quality of chronic care. Further studies might focus on addressing mismatch between health workforce and workload combined with allocation of family physician in primary care contexts. In addition, studies with more rigorous designs such as effectiveness trials or real-world implementation trials are needed to ascertain the effectiveness of training or other approaches using both patient assessment and patient outcomes as indicators.

## Supplementary Information

**Additional file 1.** Questionnaire for accessing hypertension and diabetes care.

**Additional file 2: Table A1** Standardized factor loadings from confirmatory Factor analysis for CCM. **Table A2** Standardized factor loadings from confirmatory Factor analysis for 5 A model.

## Data Availability

The questionnaire is supplementary on Additional file 1. The standardized factor loadings from confirmatory factor analysis for the chronic care model and 5A model are available on Additional file 2. The additional raw datasets generated and/or analysed during the current study are not publicly available as they contain sensitive information and data which could potentially identify participants.

## Abbreviations

CCM: Chronic care model
NCD: Non-communicable disease
PCU: Primary care unit
LMICs: Low- and middle-income countries
UHC: Universal healthcare coverage
