# Supplementary material for: Assessing a national policy on strengthening chronic care in primary care settings of a middle-income country using patients’ perspectives
Source: BMC Health Serv Res. 2021 Mar 12;21:223. doi: 10.1186/s12913-021-06220-x (PMC7953793; doi:10.1186/s12913-021-06220-x)
Supplement: Supplementary file 1 — Additional file 1. Questionnaire for accessing hypertension and diabetes care. [file 12913_2021_6220_MOESM1_ESM.docx]

**Supplement**

**Questionnaire for accessing hypertension and diabetes care**

**Based on the concept of integrated people-centered health services**

**Instruction for respondents**

Chronic diseases, especially diabetes and hypertension, are life-long patient's condition. Thus, continuity of care and patient self-management are critical for controlling blood pressure and blood glucose level.

This questionnaire was made for you to evaluate the services provided by health facilities that you registered and visit regularly. The questions include your opinion about your hypertension/diabetes care and provider team's response to address your problems and concerns. To complete this questionnaire is voluntary. If you agree to answer the survey, it will take about 10 -15 minutes. Your provided information will be confidential, and the researchers will use this information only for academic purposes. The data will not be presented individually and will not affect the respondents. The researcher team sincerely appreciates the time and effort you made to complete this questionnaire.

**Instruction for data collectors**

1. This study's populations are hypertension/diabetes patients who have registered with a health facility or live in the catchment area of a health facility.
2. This study samples are hypertension/diabetes patients who have registered with a hospital in the district or primary care cluster (Upgraded PCU) in the district, which participated in this study.
3. The sample size for each recruited district is aimed at 200 patients, and the samples are divided into two groups.
   1. First are 100 patients who live and registered with the hospital in their district. (Ratio between hypertension and diabetes patient correlate with the ration of registered patients)
   2. Second are 100 patients who live and registered with the primary care cluster, which participated in this study. (Ratio between hypertension and diabetes patient correlate with the ration of registered patients)
4. Data collectors must not collect the data in a healthcare facility but do in the patients' communities and households. After sampling the community, the data collectors collect the data from the registered patients in the community until complete the number of sample size.

**This questionnaire consists of three parts.**

Part1: Personal information of the respondents

Part2: Perception of the interactions with providers

Part3: Questionnaire about chronic care services

**Part1: Personal information of the respondents**

1. Name of a health facility that you visit regularly …………………………….

Health Facility Code ……………………..

1. Type of the health facility that you have registered and visit regularly

⬜ Hospital NCD Clinic

⬜ Trained primary care unit(upgraded PCU)

⬜ Primary care unit (upgraded PCU)

⬜ Ordinary PCU

1. Gender

⬜ Male ⬜ Female ⬜ N/A

1. Age ……… Year
2. Education attainment

⬜ Uneducated ⬜ Bachelor Degree

⬜ Primary School or Lower ⬜ Higher than Bachelor Degree

⬜ Secondary to High school

1. Occupation for the main income

⬜ None ⬜ Self-employed

⬜ Agriculture ⬜ Civil Servant

⬜ Wage Worker ⬜ Others …………….

1. Chronic condition

⬜ Diabetes ⬜ Hypertension

⬜ Both Diabetes and Hypertension

1. Duration with the chronic condition ………… Year ……………..Month
2. Last visit at the healthcare facility

Month: ……………………. Year 20……….

1. Hypertension/Diabetes care result

⬜ Controlled ⬜ Got complications

⬜ Uncontrolled

1. The latest fasting blood glucose level

Date: ……………. Result: ……………. mg/dL

(If missing, write ‘9999’ in both blanks)

1. The latest HbA1c level

Date: ……………. Result: …………….

(If missing, write ‘9999’ in both blanks)

1. The latest blood pressure

Date: ……………. Result: …………….

(If missing, write ‘9999’ in both blanks)

1. Health insurance status

⬜ Universal coverage scheme ⬜ Cash payment

⬜ Social security scheme ⬜ Other health

insurance …………….

⬜ Civil servant medical benefit scheme

**Part2: Perceptions of the interactions with providers**

1. Do you know about family doctor?

⬜ Yes ⬜ No

1. Do you know your family doctor’s name?

⬜ Yes: Name…………………… ⬜ No

1. Do you know your healthcare provider’s name?

⬜ Yes: Name…………………… ⬜ No

1. Do you have any contact channel with the healthcare provider?

⬜ No (Skip to item no.21)

⬜ Phone

⬜ Line application

⬜ Other ………………………

1. In case of you have a phone contact or Line application of your healthcare provider, have you ever contacted the provider through these channels?

⬜ Yes ⬜ No (Skip to item no.21)

1. Which issue(s) that you contact the healthcare provider if you used to use any contact channel?

⬜ For arrangement of a visit

⬜ Drug information

⬜ Consult for the illness

⬜ Complaint about the care

⬜ Other ………………………

1. Do you receive care from the same provider on repeated visits at health facilities?

⬜ Yes ⬜ No

1. Do you receive care from the same doctor on repeated visits at health facilities?

⬜ Yes ⬜ No

1. Who will be your first contact when you have the question about your health, or when you need for health-related help?

⬜ Neighbor ⬜ Family member

⬜ Family provider ⬜ Village health volunteer

⬜ Community leader/ President ⬜ Family doctor

of Local Administrative Organizations ⬜ Other ………………………

Table A1 Standardized factor loadings from confirmatory Factor analysis for CCM

| **Standardized** | **Coef.** | **Std. Err.** | **z** | **P>z** | **[95% Conf.** | **Interval]** |
| --- | --- | --- | --- | --- | --- | --- |
| Asked for my ideas when we made a treatment plan. | 0.79 | 0.01 | 92.78 | 0 | 0.77 | 0.81 |
| Given choices about treatment to think about. | 0.72 | 0.01 | 75.58 | 0 | 0.70 | 0.74 |
| Asked to talk about any problems with my medicines or their effects. | 0.47 | 0.01 | 34.26 | 0 | 0.44 | 0.49 |
| Given a written list of things I should do to improve my health. | 0.73 | 0.01 | 69.79 | 0 | 0.71 | 0.75 |
| Satisfied that my care was well organized. | 0.30 | 0.02 | 19.17 | 0 | 0.27 | 0.33 |
| Shown how what I did to take care of my illness influenced my condition. | 0.60 | 0.01 | 50.51 | 0 | 0.58 | 0.62 |
| Asked to talk about my goals in caring for my illness. | 0.73 | 0.01 | 78.21 | 0 | 0.71 | 0.74 |
| Helped to set specific goals to improve my eating or exercise. | 0.70 | 0.01 | 73.02 | 0 | 0.69 | 0.72 |
| Given a copy of my treatment plan. | 0.57 | 0.01 | 48.55 | 0 | 0.55 | 0.59 |
| Encouraged to go to a specific group or class to help me cope with my chronic illness. | 0.67 | 0.01 | 67.68 | 0 | 0.65 | 0.69 |
| Asked questions, either directly or on a survey, about my health habits. | 0.45 | 0.01 | 33.37 | 0 | 0.43 | 0.48 |
| Sure that my doctor or nurse thought about my values and my traditions when they recommended treatments to me | 0.64 | 0.01 | 60.8 | 0 | 0.62 | 0.67 |
| Helped to make a treatment plan that I could do in my daily life. | 0.79 | 0.01 | 100.79 | 0 | 0.78 | 0.81 |
| Helped to plan ahead so I could take care of my illness even in hard times. | 0.78 | 0.01 | 99.78 | 0 | 0.77 | 0.80 |
| Encouraged to attend programs in the community that could help me. | 0.61 | 0.01 | 53.27 | 0 | 0.59 | 0.63 |
| Referred to a dietitian, health educator, or counselor. | 0.76 | 0.01 | 93.55 | 0 | 0.74 | 0.77 |
| Told how my visits with other types of doctors, like the eye doctor or surgeon, helped my treatment. | 0.82 | 0.01 | 117.55 | 0 | 0.81 | 0.83 |
| Asked how my visits with other doctors were going. | 0.85 | 0.01 | 132.27 | 0 | 0.84 | 0.86 |
| Contacted after a visit to see how things were going. | 0.58 | 0.01 | 49.66 | 0 | 0.56 | 0.60 |

Table A2 Standardized factor loadings from confirmatory Factor analysis for 5 A model

| **Standardized** | **Coef.** | **Std. Err.** | **z** | **P>z** | **[95% Conf.** | **Interval]** |
| --- | --- | --- | --- | --- | --- | --- |
| Asked for my ideas when we made a treatment plan. | 0.69 | 0.01 | 68.43 | 0 | 0.67 | 0.71 |
| Asked questions, either directly or on a survey, about my health habits. | 0.41 | 0.01 | 27.79 | 0 | 0.38 | 0.44 |
| Asked how my visits with other doctors were going. | 0.69 | 0.01 | 55.82 | 0 | 0.67 | 0.72 |
| Asked what I would like to discuss about my illness at that visit. | 0.70 | 0.01 | 58.98 | 0 | 0.68 | 0.73 |
| Given a written list of things I should do to improve my health. | 0.74 | 0.01 | 77.29 | 0 | 0.72 | 0.76 |
| Shown how what I did to take care of my illness influenced my condition. | 0.57 | 0.01 | 49.23 | 0 | 0.55 | 0.60 |
| Given a copy of my treatment plan. | 0.57 | 0.01 | 49.89 | 0 | 0.55 | 0.59 |
| Told how my visits with other types of doctors, like the eye doctor or surgeon, helped my treatment. | 0.54 | 0.01 | 40.88 | 0 | 0.52 | 0.57 |
| Told how important the things I do to take care of my illness (e.g., exercise) were for my health. | 0.65 | 0.01 | 55.84 | 0 | 0.62 | 0.67 |
| Given choices about treatment to think about. | 0.63 | 0.01 | 58.55 | 0 | 0.61 | 0.65 |
| Asked to talk about any problems with my medicines or their effects. | 0.45 | 0.01 | 33.83 | 0 | 0.43 | 0.48 |
| Asked to talk about my goals in caring for my illness. | 0.76 | 0.01 | 95.66 | 0 | 0.74 | 0.78 |
| Helped to set specific goals to improve my eating or exercise. | 0.76 | 0.01 | 92.55 | 0 | 0.74 | 0.77 |
| Set a goal together with my team for what I could do to manage my condition. | 0.80 | 0.01 | 111.48 | 0 | 0.78 | 0.81 |
| Encouraged to go to a specific group or class to help me cope with my chronic illness. | 0.71 | 0.01 | 77.92 | 0 | 0.69 | 0.72 |
| Sure that my doctor or nurse thought about my values and my traditions when they recommended treatments to me. | 0.59 | 0.01 | 51.87 | 0 | 0.57 | 0.61 |
| Helped to make a treatment plan that I could do in my daily life. | 0.69 | 0.01 | 68.74 | 0 | 0.67 | 0.71 |
| Helped to plan ahead so I could take care of my illness even in hard times. | 0.74 | 0.01 | 83.73 | 0 | 0.72 | 0.75 |
| Contacted after a visit to see how things were going. | 0.59 | 0.01 | 52.06 | 0 | 0.57 | 0.61 |
| Encouraged to attend programs in the community that could help me. | 0.66 | 0.01 | 65.94 | 0 | 0.64 | 0.68 |
| Referred to a dietitian, health educator, or counselor. | 0.63 | 0.01 | 58.33 | 0 | 0.61 | 0.65 |
| Asked how my work, family, or social situation related to taking care of my illness. | 0.54 | 0.01 | 44.89 | 0 | 0.51 | 0.56 |
| Helped to make plans for how to get support from my friends, family or community. | 0.69 | 0.01 | 76.31 | 0 | 0.68 | 0.71 |
