# Supplementary material for: Assessing a national policy on strengthening chronic care in primary care settings of a middle-income country using patients’ perspectives
Source: BMC Health Serv Res. 2021 Mar 12;21:223. doi: 10.1186/s12913-021-06220-x (PMC7953793; doi:10.1186/s12913-021-06220-x)
Supplement: Supplementary file 2 — Additional file 2: Table A1 Standardized factor loadings from confirmatory Factor analysis for CCM. Table A2 Standardized factor loadings from confirmatory Factor analysis for 5 A model. [file 12913_2021_6220_MOESM2_ESM.docx]

**Table A1** Standardized factor loadings from confirmatory Factor analysis for CCM

| **Standardized** | **Coef.** | **[95% Conf.** | **Interval]** |
| --- | --- | --- | --- |
| Asked for my ideas when we made a treatment plan. | 0.79 | 0.77 | 0.81 |
| Given choices about treatment to think about. | 0.72 | 0.70 | 0.74 |
| Asked to talk about any problems with my medicines or their effects. | 0.47 | 0.44 | 0.49 |
| Given a written list of things I should do to improve my health. | 0.73 | 0.71 | 0.75 |
| Satisfied that my care was well organized. | 0.30 | 0.27 | 0.33 |
| Shown how what I did to take care of my illness influenced my condition. | 0.60 | 0.58 | 0.62 |
| Asked to talk about my goals in caring for my illness. | 0.73 | 0.71 | 0.74 |
| Helped to set specific goals to improve my eating or exercise. | 0.70 | 0.69 | 0.72 |
| Given a copy of my treatment plan. | 0.57 | 0.55 | 0.59 |
| Encouraged to go to a specific group or class to help me cope with my chronic illness. | 0.67 | 0.65 | 0.69 |
| Asked questions, either directly or on a survey, about my health habits. | 0.45 | 0.43 | 0.48 |
| Sure that my doctor or nurse thought about my values and my traditions when they recommended treatments to me | 0.64 | 0.62 | 0.67 |
| Helped to make a treatment plan that I could do in my daily life. | 0.79 | 0.78 | 0.81 |
| Helped to plan ahead so I could take care of my illness even in hard times. | 0.78 | 0.77 | 0.80 |
| Encouraged to attend programs in the community that could help me. | 0.61 | 0.59 | 0.63 |
| Referred to a dietitian, health educator, or counselor. | 0.76 | 0.74 | 0.77 |
| Told how my visits with other types of doctors, like the eye doctor or surgeon, helped my treatment. | 0.82 | 0.81 | 0.83 |
| Asked how my visits with other doctors were going. | 0.85 | 0.84 | 0.86 |
| Contacted after a visit to see how things were going. | 0.58 | 0.56 | 0.60 |

**Table A2** Standardized factor loadings from confirmatory Factor analysis for 5 A model

| **Standardized** | **Coef.** | **[95% Conf.** | **Interval]** |
| --- | --- | --- | --- |
| Asked for my ideas when we made a treatment plan. | 0.69 | 0.67 | 0.71 |
| Asked questions, either directly or on a survey, about my health habits. | 0.41 | 0.38 | 0.44 |
| Asked how my visits with other doctors were going. | 0.69 | 0.67 | 0.72 |
| Asked what I would like to discuss about my illness at that visit. | 0.70 | 0.68 | 0.73 |
| Given a written list of things I should do to improve my health. | 0.74 | 0.72 | 0.76 |
| Shown how what I did to take care of my illness influenced my condition. | 0.57 | 0.55 | 0.60 |
| Given a copy of my treatment plan. | 0.57 | 0.55 | 0.59 |
| Told how my visits with other types of doctors, like the eye doctor or surgeon, helped my treatment. | 0.54 | 0.52 | 0.57 |
| Told how important the things I do to take care of my illness (e.g., exercise) were for my health. | 0.65 | 0.62 | 0.67 |
| Given choices about treatment to think about. | 0.63 | 0.61 | 0.65 |
| Asked to talk about any problems with my medicines or their effects. | 0.45 | 0.43 | 0.48 |
| Asked to talk about my goals in caring for my illness. | 0.76 | 0.74 | 0.78 |
| Helped to set specific goals to improve my eating or exercise. | 0.76 | 0.74 | 0.77 |
| Set a goal together with my team for what I could do to manage my condition. | 0.80 | 0.78 | 0.81 |
| Encouraged to go to a specific group or class to help me cope with my chronic illness. | 0.71 | 0.69 | 0.72 |
| Sure that my doctor or nurse thought about my values and my traditions when they recommended treatments to me. | 0.59 | 0.57 | 0.61 |
| Helped to make a treatment plan that I could do in my daily life. | 0.69 | 0.67 | 0.71 |
| Helped to plan ahead so I could take care of my illness even in hard times. | 0.74 | 0.72 | 0.75 |
| Contacted after a visit to see how things were going. | 0.59 | 0.57 | 0.61 |
| Encouraged to attend programs in the community that could help me. | 0.66 | 0.64 | 0.68 |
| Referred to a dietitian, health educator, or counselor. | 0.63 | 0.61 | 0.65 |
| Asked how my work, family, or social situation related to taking care of my illness. | 0.54 | 0.51 | 0.56 |
| Helped to make plans for how to get support from my friends, family or community. | 0.69 | 0.68 | 0.71 |
